# Psychophysical responses in patients receiving a mock laser within context of an acupuncture clinical trial: an interoceptive perspective

**DOI:** 10.1186/s12906-017-1859-0

**Published:** 2017-07-03

**Authors:** Shohreh Razavy, Marcus Gadau, Shi Ping Zhang, Fu Chun Wang, Sergio Bangrazi, Christine Berle, Mahrita Harahap, Tie Li, Wei Hong Li, Christopher Zaslawski

**Affiliations:** 1 School of Chinese Medicine, Hong Kong Baptist University, 7 Baptist University Road Kowloon Tong, Hong Kong, SAR China; 2 Changchun University of Traditional Chinese Medicine, Changchun, Jilin, 130117 China; 3 Istituto Paracelso, 00153 Rome, Italy; 4 School of Life Sciences, University of Technology Sydney, Sydney, NSW 2007 Australia

**Keywords:** De Qi, Interoceptive, Acupuncture, Psychophysical responses, Mock laser

## Abstract

**Background:**

The psychophysical responses induced by verum acupuncture are characterized by a constellation of unique subjective sensory responses commonly termed De Qi. Furthermore, a variety of sham interventions have been used as a control for acupuncture clinical trials. Indeed, one such control has been mock laser which has been used as control intervention in several acupuncture clinical controlled trials. The current study aim was to examine the De Qi sensory responses and its related characteristics elicited from acupuncture and compare them to those reported following sham laser in participants enrolled in a clinical trial.

**Methods:**

The study was embedded in a multi-center, two-arm randomised clinical trial, which evaluated the effect of acupuncture on lateral elbow pain. De Qi was assessed using the Massachusetts General Hospital Acupuncture Sensation Scale (MASS). Ninety-six participants were randomly allocated to receive either acupuncture (*n* = 47) or mock laser (*n* = 49) at the acupoints LI 10 and LI 11.

**Results:**

Participants in both intervention groups reported similar De Qi psychophysical characteristics; however, both intensity and frequency of the individually perceived De Qi characteristics were significantly higher in the acupuncture group. ‘Soreness’, ‘deep pressure’, and ‘fullness-distension’ in the acupuncture group and ‘tingling’, and ‘sharp pain’ in mock laser group, were identified as the leading characteristics. Similar level of MASS De Qi Index (MDI) scores were reported for ‘Hong Kong-China’ and ‘Australia-Italy’ with a significantly higher level of De Qi reported by ‘Hong Kong-China’. Furthermore, two distinct De Qi categories were identified, namely De Qi (in line with classical sensory responses of *Suan, Ma, Zhang,* and *Zhong)* and pain.

**Conclusions:**

Subjective ‘somatic or interoceptive awareness’ should be taken into account when De Qi psychophysical responses are examined. The study accentuates the necessity and the significance of further research into interoception phenomenon which may contribute to a better understanding of the placebo effect and De Qi psychophysical responses.

**Trial registration:**

Australian and New Zealand Clinical Trial Registry reference: ACTRN12613001138774 on 11th of October 2013.

## Background

An important component of the traditional theory of acupuncture and moxibustion, De Qi [得 氣] commonly known as obtaining Qi [1–4], is considered a key element in achieving a satisfactory therapeutic effect [1, 2, 5, 6]. While the concept is affirmed in the earliest Chinese medical texts, the *Neijing* (The Yellow Emperor’s Classic of Internal Medicine) [7–10] and the *Nanjing* (The Classic of Difficulties) [11–13], details of the De Qi phenomenon, which may comprise the acupuncturist’s tactile perception of sensations and/or the patient’s experience of psychophysical responses [10, 13–16], were not fully described until recently [6, 17]. A patient’s subjective perception of De Qi is often characterised as a composite of unique psychophysical responses perceived at, or near, the site of needling and frequently typified as an assemblage of specific sensations described as “*Suan* (aching/soreness), *Ma* (tingling/numbness), *Zhang* (fullness/distension-pressure) and *Zhong* (heaviness)” [10, 18–24]. A number of attempts have been made by researchers to establish a credible rating scale to quantify De Qi such as the Acupuncture Sensation Scale [25], the Park questionnaire [26], the Subjective Acupuncture Sensation Scale [27], the MASS Scale [17], the De Qi composite [28], and the Southampton Needle Sensation Questionnaire [29]. While De Qi has been traditionally intended to describe the perceptions of the practitioner [11, 22], most of the developed scales have evaluated the patient’s perception of sensory responses.

Interestingly, although elicitation of De Qi is mostly ascribed in relation to acupuncture needle and its manipulation [30], De Qi can also be elicited without cutaneous sensory input, such as the use of laser acupuncture [16, 30]. Given that De Qi is believed to play a pivot role in the therapeutic effect of acupuncture, and may serve as an indication for “dose” of acupuncture needling [31, 32] it is essential to investigate individual De Qi characteristics induced by acupuncture.

In acupuncture research determining an appropriate control remains one of the most difficult methodological challenges [33]. The use of sham laser has been reported to serve as a valid control due to similar credibility to acupuncture and the lack of specific sensory input on the peripheral nervous system [34]. However, the induced De Qi sensory responses of acupuncture and sham laser, which may have a decisive influence on credibility, is generally considered as one of several factors that may influence the placebo response [35], has never been determined. The main objective of this study was, therefore, to examine the De Qi sensory responses and its related characteristics elicited from acupuncture and compare them to those from the administration of mock laser in participants enrolled in a clinical trial of acupuncture for lateral elbow pain.

## Methods/design

### Trial design and randomisation

Key-items of the study were designed according to the STRICTA and CONSORT statements [36, 37]. This study was embedded in a multi-site randomised, double blinded (outcome assessor and participant) controlled clinical trial, the Tennis Elbow Acupuncture-International Study-China, Hong Kong, Australia, Italy (TEA IS CHAI), to investigate the efficacy of acupuncture for the lateral elbow pain. For full details of the study design please refer to the published protocol [38]. Participants were randomly assigned to one of the two groups; Traditional Chinese acupuncture (treatment group) and inactive mock laser therapy (control group). The trial was registered with the Australian and New Zealand Clinical Trial Registry following approval from each of the four institution’s human ethics committees prior to the commencement of the study (Hong Kong: HASC/12-13/0269; Australia: HREC REF NO 2009-274A; China: CCZYFYLL2012-045; Italy: IPCIRA/105) and adhered to the Declaration of Helsinki [39].

### Participants

**Fig. 1:**
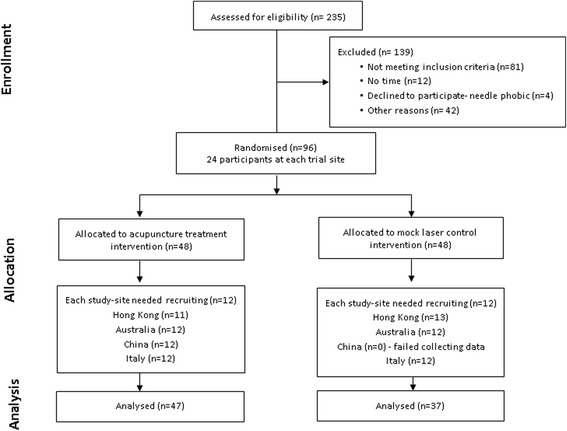
Flowchart of the trial

The current study was conducted from June 2013 till November 2014 at the outpatient clinics attached to each institution at the four study sites. Two-hundred and thirty five (*n* = 235) potential participants were invited for screening. After assessment through history taking and clinical examination for inclusion and exclusion criteria, 96 participants with a chronic Lateral Elbow Pain (LEP) commonly called tennis elbow (*n* = 24 per study site) were selected and enrolled in the trial (see Fig. 1).

Table 1 shows inclusion and exclusion criteria employed to recruit participants.

**Table 1:** Inclusion and exclusion criteria used to select participants for the clinical trial

Inclusion criteria	Exclusion criteria
Age 18-80	Central or peripheral nervous system disease
Men and women	Inflammatory rheumatic diseases
Chronic lateral elbow pain	Gout
(Duration ≥3 months)	Earlier episodes of lateral elbow pain treated surgically or with;
Unilateral localization	– Acupuncture treatment or physiotherapy for tennis elbow within the previous 3 months,
	– Acupuncture treatment for any problems within the previous week,
	– Concurrent physiotherapy for tennis elbow.

### Interventions

#### Treatment intervention

Acupuncture points were selected based on Traditional Chinese Medicine (TCM) meridian theory [40], frequently suggested acupoints for LEP [41], and agreement by all the four study sites following positive outcomes from a pilot study undertaken by one of the research groups in China [42].

Two classical acupoints, Large Intestine 11 (LI11-*Quchi*) and Large Intestine 10 (LI10-*Shousanli*), were located according to the World Health Organisation (WHO) Standard Acupuncture Point Locations in the Western Pacific Region [43]. Single-use, stainless steel, sterile, 0.30 mm × 40 mm filiform needles (Hua Tuo) were used at all sites.

**Fig. 2:**
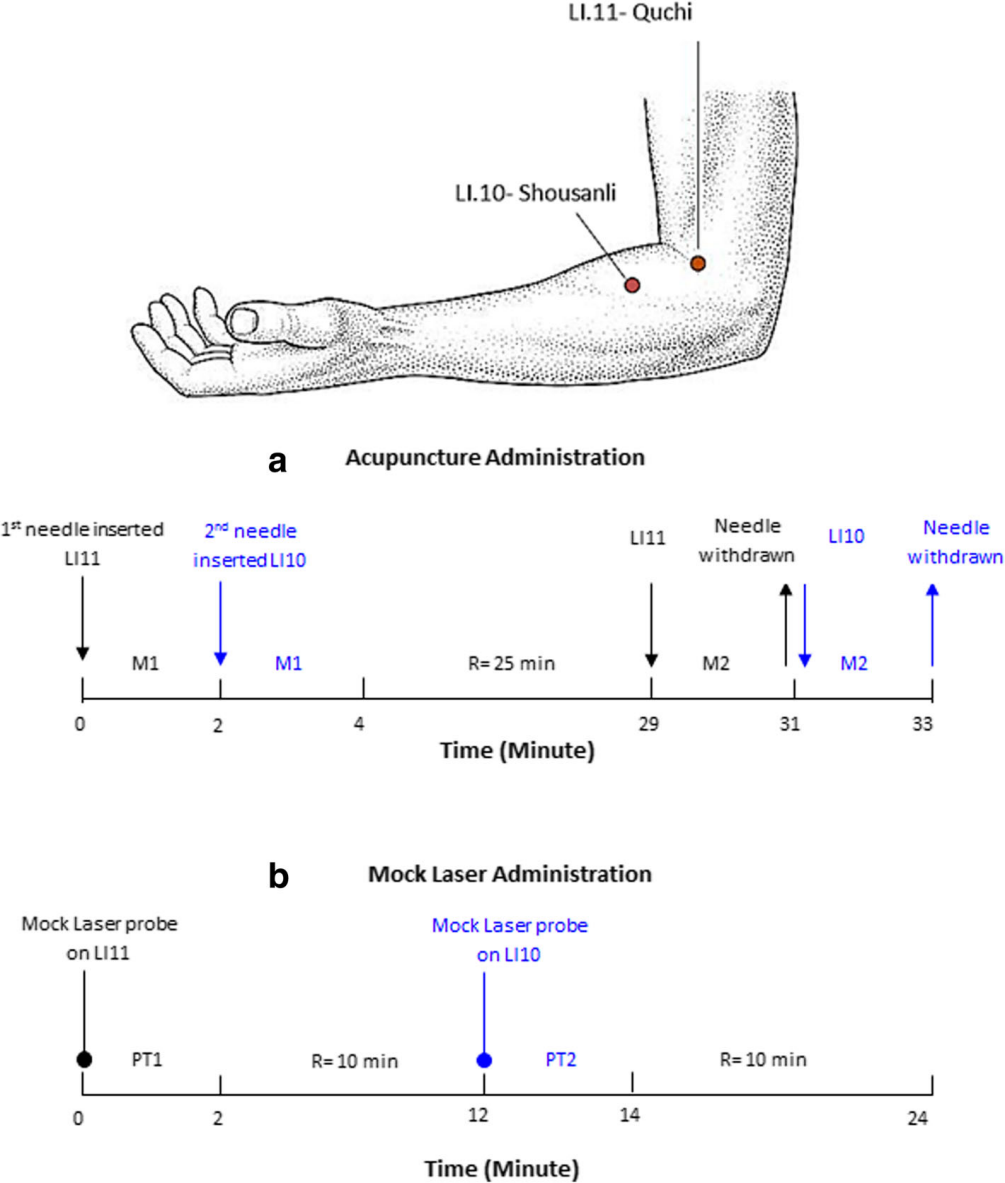
**a** indicated the manual needle manipulation at acupoint LI11 followed by LI10 on the affected side. First time Manipulation (M1); Second time Manipulation (M2); Rest period (R). **b** indicated the use of inactive mock laser probe on the same acupoints to acupuncture group at different time interval; First time Probe Touch (PT1); Second time Probe Touch (PT2); Rest period (R)

LI 11 was needled first with perpendicular deep insertion to approximately 3 cm (1.5 Cun) then the needle was withdrawn to 2 cm depth (relative to the amount of forearm muscle). Needling psychophysical response “De Qi” was sought on both acupoints by using a classical manual needle manipulation technique, “*wagging the dragon tail*” [44–46], for 2 min or to participant’s tolerance after which the needle was left in situ. The technique involved holding the needle at the end of the handle and bending the shaft of the needle 45° left and right with a speed of 1 Hz (bend both left and right within 1 s). The same two-minute manipulation procedure was applied to LI 10. In this case, however, the needle was inserted obliquely at 45 degrees pointing proximally towards the elbow. Following the stimulation of both acupoints, the needles were left for a further 24 min and then the whole manipulation procedure repeated and needles withdrawn (see Fig. 2). Nine treatments were administered in total for each intervention group, three sessions weekly over a three-week period. The practitioners administering the interventions were qualified acupuncturists with a minimum of 7 years clinical practice and were familiar with the treatment protocol.

#### Control intervention

Determining an appropriate control intervention for use in acupuncture research stays one of the leading methodological challenges acupuncture researchers encounter [33]. For acupuncture clinical trials, the ideal control intervention has not yet been determined [47] and there is, as yet, no single placebo device that can serve as the best control for all nonspecific factors involved in acupuncture treatment [33]. Furthermore, there is no agreement within the scientific research community as to which control method should be utilised [48]. In an effort to satisfy a rigorous scientific standard, mock laser is believed to be a convincing comparable application which will control for nonspecific effects such as the participant/practitioner attention and time interaction. Since the purpose of the trial initially was to investigate the effect of acupuncture in total, instead of the specificity of acupuncture regime (point specificity and needle insertion), an inactive mock laser unit was selected to be used in the control group. Although the participant could not be blind to whether they received acupuncture or laser treatment they could be blind to whether they are received an active laser treatment or not.

For the control group, an almost identical intervention procedure to the treatment group was used except that sham laser was used instead of acupuncture. The laser probe was lightly rested on the skin at the same acupoints for two-minute with a 10 min rest time in between (see Fig. 2).

To maintain the power of control procedure, visual and acoustic signals accompanied the operation of the mock laser. While different laser units were used across the four study sites, all units had the laser diode removed and a sound indicator to represent functionality.

### Outcome measure

A Credibility Rating Scale (CRS) was administered to all participants to evaluate the credibility and adequacy of the mock laser control [49], after allocation to either intervention group and just prior to the first treatment session. The scale comprised two questions as follows: (i) “How confident are you that this treatment can alleviate your complaint?”; (ii) “How logical does this treatment seem to you?”. A seven-point Likert scale rating from ‘not confident’ (0) to ‘confident’ (6) to the first question and ‘not logical’ (0) to ‘logical’ (6) to the second question was used to capture the participant’s responses. For further details please refer to the published protocol [38].

The Massachusetts General Hospital (MGH) Acupuncture Sensation Scale (MASS) [17] was used to measure the psychophysical responses elicited by the two interventions (acupuncture and mock laser). The MASS is a revised version of an earlier quantitative assessment tool, the Subjective Acupuncture Sensation Scale (SASS) [27], developed in a previously reported pilot study. The scale includes 12 descriptors (soreness, aching, deep pressure, heaviness, fullness/distension, tingling, numbness, sharp pain, dull pain, warmth, cold, and throbbing), each represented using a 10-point Likert scale rating from ‘no sensation’ (0) to ‘unbearable’ (10). The scale also comprised one blank line for participants to identify an additional sensory response if the above descriptors did not embody the sensory responses experienced during stimulation. However sharp pain was regarded to result from an inadvertent noxious stimulation rather than acupuncture De Qi [50], it was retained on the MASS as it can occur during acupuncture under certain circumstances [17].

The procedure for MASS administration was introduced by a written script according to the required published instructions [17]. Participants were required to recall any responses that they experienced during the intervention, following the two measurement sessions (session 1 and session 9), immediately after administration of each intervention.

To quantify the total intensity of De Qi experienced by each individual, the MASS De Qi Index (MDI) was calculated. The MDI is defined as the weighted average of the intensity of De Qi sensory responses elicited during the intervention using an exponential smoothing [17]. This index is considered convenient to create a single value to quantitatively summarise the full multivariate breadth and depth of acupuncture sensory responses [51].

### Analysis of data

Statistical analyses were performed using SPSS (version 22). Data distribution was evaluated by both (a) numerical methods included assessment of Z-scores for skewness and kurtosis and the Shapiro-Wilk’s test and (b) visual methods included inspection of histograms and Q-Q plots for both intervention groups throughout the whole analysis. If data were normally distributed, parametric tests were performed, otherwise non-parametric tests were used. The significance level was set at α < 0.05, two sided.

### MASS de Qi index measurement

A Wilcoxon signed rank test was conducted to compare the MDI scores, across the two measurement sessions (session 1 and session 9) for both the mock laser and acupuncture group. To evaluate the group difference, the Mann-Whitney U test was conducted. If data distribution were similarly shaped, medians were compared otherwise mean ranks were used as a substitute score. Additionally, a Kruskal-Wallis H test was conducted to evaluate the distribution of the MDI scores across the four trial sites for the individual treatment group. The distribution of the scores was then assessed by visual inspection of the boxplots. Pairwise comparison was conducted using a Bonferroni correction for multiple comparisons.

### Individual de Qi psychophysical responses

#### Frequency of individual de Qi characteristics

Fisher’s Exact test was performed to investigate the occurrence of the individual De Qi characteristics between the two treatment groups as well as between the trial sites in each study groups on the data pooled from the time factor (measurement sessions).

#### Intensity of individual de Qi characteristics

A Mann-Whitney U test was conducted, comparing intensity of the individual De Qi characteristics between the two study groups. If the distribution were similarly shaped, medians were compared otherwise mean ranks were used as a substitute score.

#### Clustering of individual de Qi characteristics

To further investigate the clustering of the individual De Qi psychophysical responses for each treatment intervention, Principle Component Analysis (PCA) was applied.

## Results

Normality tests revealed that the majority of the data was not normally distributed. Most of the data including the MDI scores (continuous measure) and the individual De Qi responses, measured using the MASS Likert scale (ordinal), did not comply with the assumptions of parametric tests. Since the data could not be transformed to meet the assumptions of normality, the non-parametric tests for data analysis were performed.

Of the 96 eligible participants, twelve were excluded. This was due to the fact that one trial site did not administer the MASS to the mock laser participants (*N* = 12), resulting in unequal numbers in each group (acupuncture 47 cf. mock laser 37). Accordingly, the MASS questionnaire was administered to 84 participants, the acupuncture group (*N* = 47) and the mock laser group (*N* = 37), at the two measurement sessions.

The result of the CRS, conducted prior to commencing the intervention phase, demonstrated no significant difference between the two study groups for both expectancy of complaint’s improvement (*p* = 0.772) and rational for treatment (*p* = 0.768), implying that the control intervention was perceived as credible and adequate in the current study setting.

**Fig. 3:**
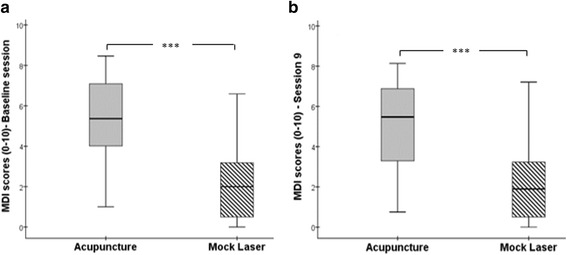
**a** and **b** MASS De Qi Index (MDI) scores for the treatment and control group at session 1 and session 9 respectively, (*n* = 47 for the treatment group, *n* = 37 for the control group). The boxes are bound by the interquartile range (IQR) (top of the box represents the 75th percentile, while the bottom of the box represents the 25th percentile). The boxes are divided by the median, and the whiskers attached to the box represent the minimum and maximum scores. ***: statistically significant difference (*p* < 0.001) between the two groups

The result showed that the participant’s perceptions of De Qi were similar at the two measurement sessions for both the acupuncture group (Z = −1.76, *p* = 0.079) and the mock laser group (Z = −0.85, *p* = 0.932). By contrast, evaluation of the MDI scores showed a statistically significant difference between the two groups for each measurement session. Participants in the acupuncture group reported statistically significantly higher levels of De Qi (*mean rank = 59.02*, interval range = 1-8.47) compared to participants in the mock laser group (*mean rank = 23.18*, interval range = 0-6.59) at session 1 (*U* = 140, *p* < 0.001). At session 9, a similar response was also observed between the two groups (*U* = 284.50, *p* < 0.001) with MDI scoring higher (*mean rank = 55.95*, interval range = 0.75-8.14) in the acupuncture group than in the mock laser group (*mean rank = 26.99*, interval range = 0-7.21) (see Fig. 3a and b).

The results indicated that while participants in the mock laser group perceived some acupuncture psychophysical responses, the experienced sensory responses in the acupuncture group was significantly more intense than in the mock laser group.

A Kruskal-Wallis H test was run to determine if there were any differences in the MDI scores across the four study centres; ‘Hong Kong (HK)’, ‘Australia (AUS)’, ‘China (CHA)’, and ‘Italy (ITY)’ in each treatment group. Time factor for each study site in each intervention group was investigated and since there was not any statistically significant difference between the scores, the data for 2 weeks were pooled together.

**Fig. 4:**
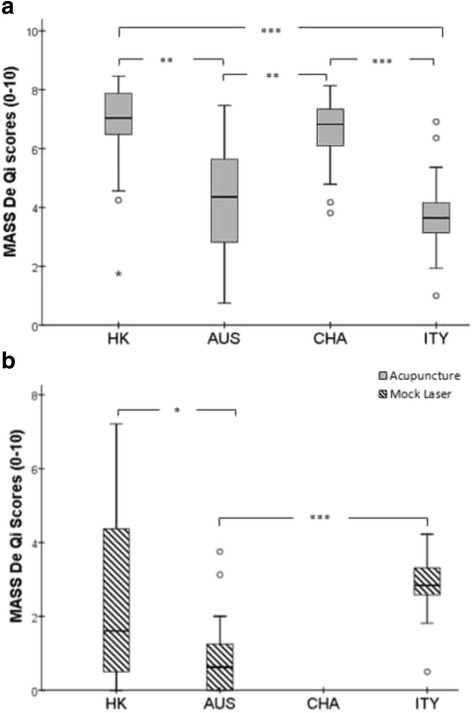
**a** and **b** MASS De Qi difference among trial sites for the acupuncture group a and the mock laser group b individually. The box plots demonstrate comparison of the MASS De Qi median scores between the four trial sites for both the acupuncture group (HK = 22, AUS = 24, CHA = 24, ITY = 24) and the mock laser group (HK = 26, AUS = 24, ITY = 24). One trial site (China) did not collect data for the mock laser group. Bonferroni correction for multiple comparisons was used. Pairwise comparison demonstrated statistically significant differences in the MDI scores across different trial sites (Kruskal-Wallis Rank Sum test, * *p* < 0.05, **p < 0.01, ***p < 0.001; 2-tailed). Extreme values and outliers lied beyond the whiskers and denoted differently with a star and a circle respectively

As the distribution of MDI scores was dissimilar for all the study sites, assessed by visual inspection of a boxplot, the mean rank was reported. The result showed that in the acupuncture group the MDI mean rank was statistically significantly different between the trial sites (Χ^2^(3) = 41.86, *p* < .001). In mock laser group, a similar trend was also observed (Χ^2^(2) =20.65, *p* < 0.001). Pairwise comparisons were performed using Dunn’s (1964) procedure with a Bonferroni correction for multiple comparisons and adjusted *p*-values are presented. The post hoc analysis revealed statistically significant differences in MDI scores between ITY (mean rank = 24.38) and HK (*mean rank* = 67.32) (*p* < 0.001), ITY and CHA (*mean rank* = 63.96) (*p* < 0.001), AUS (*mean rank* = 36) and HK (*P* = 0.001), and AUS and CHA (*P* = 0.002); but not between other trial site combination (AUS-ITY) and (CHA-HK). In mock laser control group, the post hoc showed statistically significant differences in MDI scores between AUS (*mean rank* = 23.02) and HK (*mean rank* = 38.33) (*p* = 0.035), AUS and ITY (*mean rank* = 51.08) (*p* < 0.001), but not between the HK and ITY group combination (see Fig. 4a and b).

Following administration of acupuncture, participants reported various De Qi psychophysical responses. Interestingly participants in the control group who received mock laser reported similar sensory responses to the acupuncture group. Yet, comparison of the individual De Qi characteristics demonstrated statistically significantly higher frequency in the acupuncture group versus the mock laser group (*p* < 0.001) with approximately a similar psychophysical response profile (see Table 2).

**Table 2:** Comparison of the frequency of individual De Qi characteristics between the two study groups

De Qi characteristics	Acupuncture		Mock Laser		Fisher’s Exact p
	Count	%	Count	%	
1.Soreness	87	92.5	32	43.9	.000^***^
2.Aching	87	92.5	34	45.9	.000^***^
3.Deep Pressure	81	86.0	30	40.6	.000^***^
4.Heaviness	76	80.8	29	39.2	.000^***^
5.Fullness/Distention	76	80.8	27	36.1	.000^***^
6.Tingling	66	70.2	37	50.0	.004^**^
7.Numbness	73	78.5	32	43.2	.000^***^
8.Sharp Pain	57	60.7	33	44.6	.002^**^
9.Dull Pain	57	60.6	26	36.2	.000^***^
10.Warmth	32	34.0	14	19.2	.020^*^
11.Cold	47	50.0	15	20.4	.000^***^
12.Throbbing	27	35.5	8	11.0	.001^**^

In all cases the expected frequencies were less than five in each cell and therefore Fisher Exact test was displayed, * p<0.05, **p <0.01, ***p <0.001

**Fig. 5:**
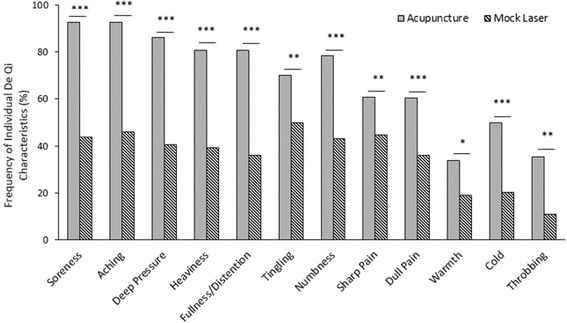
Comparison of the frequency of individual De Qi psychophysical responses during acupuncture and mock laser intervention. Data for weeks were pooled together. Frequencies calculated upon the number of participants reporting perceptions within each study arm (*n* = 47 for the acupuncture group, *n* = 37 for the mock laser group). Data related to mock laser group (Centre 3) was excluded from data analysis. Each De Qi characteristic was shown on a Likert scale rating (0-10); In all cases the expected frequencies were less than five in each cell and therefore Fisher Exact test was used, * *p* < 0.05, **p < 0.01, ***p < 0.001

The frequency of De Qi responses was compared between the two study groups. The data was analysed as a binary indication of presence and absence and a sensory response was presented if the reported level reached a minimum score of one. So, the presence and absence (no sensation) was tabulated for the individual 12 De Qi characteristics with acupuncture group versus mock laser group. Descriptively the two intervention groups differed in how often these qualities were selected to characterise perceptions. Among the psychophysical responses recorded within group; ‘Soreness’ and ‘Aching’ (92.5%), ‘Deep pressure’ (86%), ‘Fullness/ Distension’ and ‘Heaviness’ (80.8%), for acupuncture and ‘Tingling’ (50%), ‘Aching’ (45.9%), and ‘Sharp pain’ (44.6%) for mock laser were rated as the three leading qualities among all the trial sites (see Fig. 5). Among the 12 sensory responses, perhaps ‘Tingling’ could be considered as the best discriminator between acupuncture and the tactile mock laser control since this response was not shared among the two study groups.

Frequencies of the adjectives used to characterise perceptions during the acupuncture and mock laser treatment among the four trial sites were also investigated, and are displayed in Table 3. Except for ‘cold’ and ‘throbbing’ where there were no statistically significant differences in the reported frequency across the study sites in the mock laser group, other De Qi characteristics demonstrated statistically significant difference in frequency across the different trial sites for each treatment group (*p* < 0.001).

**Table 3:** Comparison of the individual De Qi characteristics across the study sites between the two study groups

	Centre1				Centre2				Centre 3^Ɨ^		Centre 4							
	Acupuncture		M. Laser		Acupuncture		M. Laser		Acupuncture		Acupuncture		M. Laser		Fisher’s Exact Test			
De Qi Characteristics	*N*	%	*N*	%	*N*	%	*N*	%	*N*	%	*N*	%	*N*	%	Χ^2^_Acup._	*p-value*	Χ^2^_Laser_	*p-value*
1. Soreness	22	99.5	13	50	21	87.5	1	4.2	24	91.6	20	83	18	78.3	45.4	.000^***^	34.6	.000^***^
2. Aching	22	100	10	38.4	24	95.8	2	8.3	22	91.7	20	83.4	22	91.7	44.6	.000^***^	40.7	.000^***^
3. Deep Pressure	22	100	11	46.1	21	87.5	2	8.3	22	100	14	58.3	16	66.7	26.7	.001^**^	22.1	.000^***^
4. Heaviness	22	100	9	34.6	19	79.2	3	12.5	24	100	11	45.9	17	70.9	36.6	.000^***^	25.2	.000^***^
5. Fullness/Dis.	22	100	13	50	15	62.5	2	8.3	24	100	15	62.6	12	50	48	.000^***^	18.4	.001^**^
6. Tingling	22	100	12	46.1	17	70.9	9	37.5	14	58.4	13	52.2	16	66.7	37.9	.000^***^	12.2	.010^*^
7. Numbness	21	100	16	61.5	14	58.4	3	12.5	22	91.7	16	66.7	13	54.2	37.7	.000^***^	14.9	.002^**^
8. Sharp Pain	19	86.4	10	38.5	20	83.4	3	12.5	3	12.5	15	62.5	20	83.3	48.2	.000^***^	26.4	.000^***^
9. Dull Pain	18	79.8	11	45.9	20	83.3	2	8.3	1	4.2	18	75	13	54.2	47.6	.000^***^	18.8	.001^**^
10. Warmth	11	50	5	20	15	62.5	9	37.5	0	0	6	25	0	0	32.3	.000^***^	17.5	.000^***^
11. Cold	22	100	7	26.8	11	45.8	3	12.5	2	8.3	12	50	5	20	57.9	.000^***^	4.4	.786
12. Throbbing	8	66.6	3	11.5	13	54.2	5	20.8	1	4.2	5	31.3	0	0	29.2	.000^***^	6.7	.080

When data related to measurement sessions were pooled, virtually frequency of every single sensory characteristic demonstrated statistically significant across different study sites in each treatment group (acupuncture and mock laser). N = Number of participants reported sensory perception; Χ^2^_Acup_ = Fisher Exact test for acupuncture group; Χ^2^_Laser_ = Fisher Exact test for mock laser group; Ɨ indicates exclusion of the mock laser group (*n* = 12) from study centre 3. In all cases the expected frequencies were less than five in each cell and therefore Fisher Exact test was used, * *p* < 0.05, **p < 0.01, ***p < 0.001

In the acupuncture group, while all the participants from HK reported ‘Tingling’, ‘Numbness’, ‘Cold’, ‘Aching’, ‘Deep pressure’, ‘Heaviness’, and ‘Fullness/ Distension’ as part of their sensory perception, in CHA only the three latter characteristics were reported by all the participants. In AUS and ITY, ‘Aching’ (95.8%; 83.4%) and ‘Soreness’ (87.5%; 83%) were identified as the two shared foremost qualities respectively. By contrast in the mock laser group, different psychophysical response profiles were demonstrated when the three leading characteristics were compared among the three trial sites.

The intensity of the individual De Qi characteristics among the two study groups was also investigated using a Mann-Whitney U test. The result indicated a statistically significant difference in the intensity of the individual De Qi characteristics across the two treatment groups. Participants in the acupuncture group reported a statistically significantly higher level of De Qi characteristics compared to participants in the mock laser group at all times (*p* < 0.001), except for ‘sharp pain’ (*p* < 0.01) and ‘warmth’ which were statistically less significant (*p* < 0.05) (see Table 4).

**Table 4:** Comparison of the intensity of individual De Qi characteristics between the study groups

De Qi Psychophysical Characteristics	Acupuncture				Mock Laser					
	N*	Interval range	Mdn	Mean Rank	N*	Intervalrange	Mdn	Mean Rank	*U*	*p value*
1. Soreness	nil	0 - 9	4	109.1	25	0 - 6	0	51.7	1076.5	.000^***^
2. Aching	nil	0 - 8	2	103.6	24	0 - 7	0	60.1	1678.5	.000^***^
3. Deep Pressure	nil	0 - 8	3	107.3	24	0 - 6	0	55.5	1331.5	.000^***^
4. Heaviness	nil	0 - 8	2	104.9	24	0 - 6	0	58.5	1552.0	.000^***^
5. Fullness/ Distension	nil	0 - 9	3	105.7	24	0 - 7	0	57.5	1484.5	.000^***^
6. Tingling	nil	0 - 9	1	96.01	24	0 - 6	0	69.8	2396.0	.000^***^
7. Numbness	1	0 - 9	2	103.1	24	0 - 5	0	59.9	1660.5	.000^***^
8. Sharp Pain	nil	0 - 8	1	93.1	24	0 - 5	0	73.6	2669.5	.006^**^
9. Dull Pain	nil	0 - 8	1	95.1	26	0 - 9	0	68.4	2294.0	.000^***^
10. Warmth	nil	0 - 8	0	89.9	25	0 - 8	0	76.3	2870.5	.022^*^
11. Cold	nil	0 - 9	0	96.1	24	0 - 7	0	69.7	2382.0	.000^***^
12. Throbbing	18	0 - 7	0	82.9	27	0 - 7	0	64.4	2015.0	.000^***^

Comparison of individual De Qi qualities mean ranks among the study groups using Mann-Whitney U test (* *p* < 0.05, **p < 0.01, ***p < 0.001); N* = number of missing data, Mdn = Median; Mock laser group (Centre 3) was also excluded for analysis

**Fig. 6:**
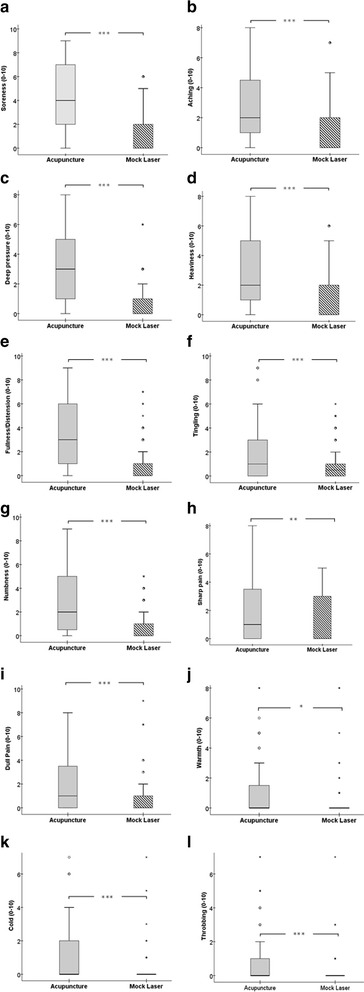
**a**-**l** Comparison of individual De Qi characteristics across the two study groups (acupuncture and mock laser). MASS De Qi score for the acupuncture and mock laser group at the session 1 and session 9, (*n* = 47 for the acupuncture group, *n* = 38 for the mock laser group). The boxes are bound by the interquartile range (IQR) (top of box represents the 75th percentile, while the bottom of the box represents the 25th percentile). The boxes are divided by the median, and the whiskers attached to the box represent the minimum and maximum scores. Extreme values and outliers lied beyond the whiskers and denoted differently with a star and a circle respectively

Distribution of individual De Qi characteristics was also investigated using a boxplot. Because of dissimilar distribution shape of individual De Qi characteristics across the two treatment groups, the mean rank is reported. Median values however, displayed in the Table 3, are indicated as being more representative of the data. Due to the high frequency of zero scores, the median values for mock laser group were identified as zero which reflects the true inactive nature of the mock laser. In most cases, boxplots related to the control mock laser group showed several outliers and/ or extreme values resulted in an untrustworthy interval range compared to the acupuncture treatment group (see Fig. 6a-l). Among all the reported characteristics, ‘soreness’ (mean rank = 109.1), ‘deep pressure’ (mean rank = 107.3), and ‘fullness/ distension’ (mean rank = 105.7) were rated as the three leading intensities among the four trial sites. In the control group, however, a different psychophysical response profile was observed, with ‘warmth’ (mean rank = 76.3), ‘sharp pain’ (mean rank = 73.6), and ‘tingling’ (mean rank = 69.8) rated as the three foremost intensities.

In general, significant differences were found in the intensity and the prevalence (frequency) of individual De Qi characteristics between the acupuncture group and the mock laser group. Both intensity and frequency of individual De Qi characteristics were significantly higher in the acupuncture group than in the mock laser group. When both the intensities and frequencies of individual De Qi characteristics were compared, ‘soreness’, ‘deep pressure’, and ‘fullness/ distension’ appeared to be the important characterisation of De Qi in acupuncture whereas ‘tingling’ and ‘sharp pain’ seemed to represent De Qi in the mock laser group.

To further investigate the structure of De Qi characteristics, Principle Component Analysis (PCA) was applied. Similar research [25, 27, 52–54] has performed PCA on similar data to explore the underlying factors and support the use of PCA as an appropriate statistical method for analysing acupuncture sensory responses under certain conditions [17]. The data were checked for both statistical (normality, linearity between all variables using a correlation matrix) and conceptual (selection of variables and homogeneity) alignment.

Two conventional criteria, eigenvalues, and scree plot, for determining the number of components were used. The eigenvalue is directly related to the amount of information contained in its associated principal component. Eigenvalues of 1 or greater are considered to be significant. The scree plot was also used to identify the optimum number of components that can be extracted before the amount of unique variance begins to dominate the common variance structure [55].

Varimax rotation has achieved the most widespread use as it seems to give the clearest separation of factors [25, 53, 54], however, oblique rotation with Kaiser normalisation was applied to the individual De Qi characteristics for the following reasons. When varimax rotation was used, it still remained a complex structure; namely, some components loading on the same individual variables were making interpretation difficult. To achieve a simple structure, so that each variable has only one component that loads strongly on it and each component loads strongly on at least three variables, oblique rotation with factor loading cut-off of 0.3 was used. Oblique rotation often represents the clustering of variables more accurately by allowing for correlated factors. It is, therefore, more realistic to assume that influences in nature are correlated at the theoretical level. [55] The pattern matrix was also used in interpreting the oblimin-rotated matrix as indicates the uncontaminated correlations between variables and factors [55].

In both the acupuncture and mock laser groups, two components with eigenvalues greater than 1.0 were identified counting in total for 64.6% and 63.27% respectively of the variance explained. Visual inspection of the scree plots in each study group also led to the retention of two components.

In the acupuncture group, the Kaiser-Meyer-Olkin (KMO) measure was 0.772 suggesting “middling” on Kaiser’s (1974) classification of measure values that is indicated for the adequacy of the sample. Bartlett’s test of sphericity was significant (Χ^2^ (66) = 550.7, *p* < .001), indicating the data was also suitable for Factor Analysis (FA). In the mock laser group, the KMO measure was 0.689 meaning “mediocre” on Kaiser’s (1974) classification of measure values. The KMO value can range from 0 to 1, with values above 0.6 suggested as a minimum requirement for sampling adequacy. Bartlett’s test of sphericity was significant (Χ^2^ (66) = 589.56, *p* < .001) indicating the data was also suitable for FA.

Investigation of pattern matrix with oblique rotation in each intervention group also showed that all the individual De Qi characteristics loaded on a single factor demonstrating a simple structure of the De Qi sensory responses (see Table 5). In the acupuncture group, ‘dull pain’, ‘sharp pain’, ‘throbbing’, ‘cold’, ‘tingling’, warmth’, and ‘aching’ were revealed as the loaded first factor. Some previous reviewed studies have identified, ‘sharp pain’ [2, 28], ‘throbbing’ [56], ‘tingling’ [56], and ‘aching’ [25, 56] as part of the pain dimension that may be caused by skin piercing and a biochemical reaction of local tissue damage [56]. Two other characteristics, ‘warmth’ and ‘cold’, based on ancient Chinese Medicine literature (*Huang Di Neijing Suwen*-chapter 54) represent the subject’s different state of illness [17]. In our study, therefore, we would like to suggest the first component as a pain dimension. The second loaded factor, ‘fullness/ distension’, ‘soreness’, ‘deep pressure’, ‘heaviness’, and ‘numbness’ as supporting the De Qi dimension-the particular constellation of subjective psychophysical responses [25, 50, 57, 58] that supposedly and are recurrently identified as the following terms; *Suan*, *Ma*, *Zhang* and *Zhong* at classical acupoints in various literature.

**Table 5:** Factor loading of De Qi perception of sensory responses in the two study groups

De Qi Characteristics	Acupuncture			Mock laser	
	C 1	C2	De Qi Characteristics	C 1	C 2
Dull pain	.857		Heaviness	.914	
Sharp pain	.823		Aching	.887	
Throbbing	.770		Deep pressure	.857	
Cold	.761		Soreness	.836	
Tingling	.728		Fullness/ Distension	.766	
Warmth	.697		Warmth	.727	
Aching	.575		Tingling	.668	
Fullness/ Distension		−.912	Sharp pain	.614	
Soreness		−.899	Numbness	.511	
Deep pressure		−.847	Throbbing		.889
Heaviness		−.781	Cold		.847
Numbness		−.664	Dull pain		.598

Explorative Principle Component Analysis (PCA) with oblique rotation in each study group produced two components represented as C1 and C2 in the table. All participants were included in the analysis and data for weeks (measurement sessions) were pooled together

In the mock laser group, PCA disclosed the two distinct components in which both dimensions namely, De Qi and pain, integrated together which made it difficult to interpret.

## Discussion

The study provided quantitative data on several features of manual acupuncture and mock laser that can be broadly specified as follows; the intensity of MASS De Qi represented as MDI scores, the prevalence, and intensity of individual De Qi characteristics, and finally the clustering of individual De Qi characteristics together for each intervention group.

The intensity of De Qi psychophysical and neurological response is proposed to serve as a basis for acupuncture dosage measurement [20, 31, 59]. The result of our study indicated that De Qi and its related characteristics can be maintained at a similar level across several measurement sessions when a standardised needling technique is applied. The capabilities of the MASS De Qi measurement scale as a surrogate measure of stimulation intensity was also demonstrated in the current study. Our results will facilitate future studies which aim to determine the ‘dose’ of acupuncture using neurophysiological approaches.

Many of the sensory responses encompassing De Qi in acupuncture also occurred in mock laser, but at a significantly lower frequency and intensity and with a disparate psychophysical response profile. ‘Soreness’, ‘deep pressure’, and ‘fullness/ distension’ reported during acupuncture and ‘tingling’, and ‘sharp pain’ reported during administration of the mock laser control were identified as the leading characteristics in which ‘tingling’ may be indicative of a difference between the two interventions.

Similar to the previous clinical research studies [16, 34], the current study provided experimental evidence indicating that De Qi can be elicited without needle insertion. Other research groups further suggest that De Qi might be a central phenomenon of bodily self-awareness and consciousness [16, 34, 60]. Shifting attention to specific body sites due to induced sensory input interacting with central brain processing may cause the perception of De Qi, and at the same time modulate cognitive and affective responses in the brain [60, 61]. It is therefore likely that during the application of the tactile mock laser the patient’s detection of self-relevance from exteroceptive (resting the probe on the skin) and interoceptive inputs (being asked about perceived sensory responses- ‘self-appraisal’ processes [62, 63]) may trigger a cascade of cortical and subcortical processing that positions the patients to perceive an increased response potential [62]. Indeed, non-specific effects due to the participant’s expectancy [16, 61, 64, 65] or experience [64], focusing (e.g. visual attention) [16, 66], general treatment settings [16] may all have a significant influence in perception of De Qi. The patient’s expectations or anticipation are partially based on self-relevant phenomena and self-referential introspection and constitute the preference [62, 63].

Interoceptive awareness [67, 68] that is characteristically personalised as the frequency of reporting bodily sensations due to body awareness and self-consciousness [66], and beliefs about the importance of such bodily states [61, 69] may partially explain the psychophysical responses reported by the participants receiving the mock laser.

Whilst acupuncture can be considered an ‘interoceptive stimulus’ as it stimulates small diameter fibres and ergoreceptors, should mock laser be considered as ‘self-referential interoceptive’ due to involvement of different cognitive and affective processes? Perhaps the idea is appropriate when different mock laser control groups are utilised in controlled trials.

The results of our study showed that a significantly higher level of De Qi was perceived in East-Asian (HK-CHA) countries in the acupuncture group. This may imply some cross-cultural similarities in the perception of De Qi and/or somatic awareness [69] induced by acupuncture only. Alternatively, it may be due to an acceptance of greater De Qi sensation by the Asian population [20].

We attempted to separate acupuncture sensory responses into two major categories, De Qi (fullness/ distension, soreness, deep pressure, heaviness, numbness) that is consistent with classical sensory responses and pain (dull pain, sharp pain, throbbing, cold, tingling, warmth, aching). Although ‘dull pain’, as opposed to ‘sharp pain’, is reported as a De Qi characteristic that is beneficial to treatment [10, 20], in our study ‘dull pain’ was clustered with other pain characteristics. While different research groups utilised ‘dull’ [2, 25, 26, 70, 71], or ‘dull ache’ [29, 72, 73] as one of the selected descriptor when developing a De Qi measurement scale, in MASS [17] ‘dull pain’ was the term utilised. Combining the two dimension characteristics, namely ‘dull’ and ‘pain’, a noxious response, would be improper when the measurement of the participant’s De Qi is sought. Participants may be misled when describing their perceived De Qi sensory responses which will, in fact, contribute to questionable content validity of the MASS scale- *the extent to which the elements within a measurement procedure are relevant and representative of the construct that they will be used to measure* [74]- of the MASS scale questionable. Since content validity affects the clinical inferences that can be drawn from the obtained data [74], we would like to suggest separating the ‘dull pain’ characteristic in which the two dimensions were melded into two separate dimension characteristics.

### Limitation and suggestion for future research

In our study, the mock laser was not truly inert. The laser probe was physically in contact (touch) with the skin surface at the two classical-acupoints for two minutes. Additionally, we cannot discount that due to operator hand fatigue some downward pressure may have been applied inadvertently. While the results from a study demonstrated activation of brain visual cortex following laser irradiation on the BL-67 acupoint, no such response was found when the switched-off laser was placed in contact with the skin at the acupoint [75]. This is in contrast with our study in which participants reported a certain degree of psychophysical responses. Light touch of the skin stimulates mechanoreceptors associated with slow conducting unmyelinated C-afferents [76–78] not only resulting in activity in the insular region but also suggested to induce a ‘limbic touch’ response leading to emotional and hormonal reactions [62, 77]. For future studies, it is therefore suggested to provide sufficient distance between skin and laser to prevent tactile stimulation. It is also recommended to recruit naïve participants to further investigate the interoceptive response.

Additionally, due to the small sample size in each trial site caution should be made in interpreting the results from group differences among the four trial sites. It should be also noted that different trial sites used several translated versions of the MASS [17, 52] and the accurate translation of particular descriptors may have been interpreted differently.

## Conclusion

In summary, our study found participants in both study groups reported characteristics in conformity with reported descriptions of classical De Qi responses. The sensory constituent of De Qi is complex to study due to the subjective nature of De Qi and the fact that it is influenced by a variety of factors, such as patient’s psychosocial context, the severity of the ailment, the location of acupoints, and the needling techniques. Patient’s expectancy/anticipation, awareness, attention to the acupoints/certain area of the body, and the general treatment settings may play an important role in the elicitation of psychophysical responses. Such results highlight the difficulty of developing a truly inert placebo intervention to mimic both the visual appearance and the needling method involved, especially for acupuncture research. Increasing research into interoception may contribute to a better understanding of the placebo effect and its psychophysical responses that are often observed in controlled trials. Both identification and differentiation of descriptors into two clusters (De Qi and pain) would facilitate the specific qualities of De Qi and control for it in experimental studies.

## Abbreviations

AUS: Australia
CHA: China
CRS: Credibility Rating Scale
FA: Factor Analysis
HK: Hong Kong
ITY: Italy
KMO: Kaiser-Meyer-Olkin
LEP: Lateral Elbow Pain
MASS: Massachusetts General Hospital Acupuncture Sensation Scale
MDI: MASS De Qi Index
PCA: Principle Component Analysis
SASS: Subjective Acupuncture Sensation Scale
TCM: Traditional Chinese Medicine
TEA IS CHAI: Tennis Elbow Acupuncture- International Study- China, Hong Kong, Australia, Italy
WHO: World Health Organisation.
